# Structure-Guided Recombination Creates an Artificial Family of Cytochromes P450

**DOI:** 10.1371/journal.pbio.0040112

**Published:** 2006-04-11

**Authors:** Christopher R Otey, Marco Landwehr, Jeffrey B Endelman, Kaori Hiraga, Jesse D Bloom, Frances H Arnold

**Affiliations:** **1**Biochemistry and Molecular Biophysics, California Institute of Technology, Pasadena, California, United States of America; **2**Division of Chemistry and Chemical Engineering, California Institute of Technology, Pasadena, California, United States of America; **3**Bioengineering, California Institute of Technology, Pasadena, California, United States of America; Brandeis UniversityUnited States of America

## Abstract

Creating artificial protein families affords new opportunities to explore the determinants of structure and biological function free from many of the constraints of natural selection. We have created an artificial family comprising ˜3,000 P450 heme proteins that correctly fold and incorporate a heme cofactor by recombining three cytochromes P450 at seven crossover locations chosen to minimize structural disruption. Members of this protein family differ from any known sequence at an average of 72 and by as many as 109 amino acids. Most (>73%) of the properly folded chimeric P450 heme proteins are catalytically active peroxygenases; some are more thermostable than the parent proteins. A multiple sequence alignment of 955 chimeras, including both folded and not, is a valuable resource for sequence-structure-function studies. Logistic regression analysis of the multiple sequence alignment identifies key structural contributions to cytochrome P450 heme incorporation and peroxygenase activity and suggests possible structural differences between parents CYP102A1 and CYP102A2.

## Abstract 2

Fragments from three cytochrome P450 proteins were recombined to produce approximately 3,000 artifical proteins. Their heme-binding ability was determined, and regression analyses on sequence alignments enabled elucidation of key structural components.

## Introduction

Our understanding of how protein sequence relates to structure and function is aided by comparisons of sequences related by evolution [ 1, 2]. With only limited numbers of highly divergent sequences, however, such analyses are often uninformative. Furthermore, because the sequences have been culled by natural selection, relationships between sequence and physical or chemical properties not under direct selection are difficult or impossible to discern. We would like to create artificial protein families in order to probe the range of sequence and functional diversity that is compatible with a given structure, free from the constraint of having to function in the narrow context of the host organism. These artificial sequences would help us to identify connections to functions that may not be important biologically (e.g., high thermostability, new substrate specificity, or ability to fold into a particular structure, but not catalyze a particular reaction), but are critical for understanding the proteins themselves [ 3, 4].

The products of millions of years of divergence and natural selection, protein families contain members that differ at large numbers of amino acids residues. Creating numerous diverse and folded sequences in the laboratory is challenging, due in part to the sparsity of proteins in sequence space. Among random sequences, estimates of the frequency of functional proteins range from 1 in 10 ^11^ [ 5] to as little as 1 in 10 ^77^ [ 6]. Randomly mutating a functional parent sequence improves the odds, but highly mutated sequences are still exceedingly unlikely to fold into recognizable proteins [ 7, 8]. The methods by which novel proteins have been created, including selection from libraries of random [ 5] or patterned [ 9] sequences, evolution from existing sequences by iterative mutation or recombination [ 10], and by structure-guided design [ 11] as well as computation-intensive protein design [ 12, 13], either yield small numbers of characterized sequences or numerous sequences with low diversity (few sequence changes).

We are developing site-directed, homologous recombination guided by structure-based computation (SCHEMA) [ 14– 16] to create libraries of protein sequences that are simultaneously highly mutated and have a high likelihood of folding into the parental structure. Mutations made by recombination of functional sequences are much more likely to be compatible with the particular protein fold than are random mutations [ 17]. SCHEMA calculations allow us to minimize the number of structural contacts that are disrupted when portions of the sequence are inherited from different parents, further increasing the probability that the chimeric proteins will fold. The validity of the SCHEMA disruption metric has been demonstrated in previous work [ 14– 16]. SCHEMA, however, has not yet been used to design a library to maximize the number of sequences with low disruption and high mutation.

Here we report SCHEMA-guided recombination of three cytochromes P450 to create 6,561 chimeras, of which ˜3,000 are properly folded P450 proteins. Cytochromes P450 comprise a superfamily of heme enzymes with myriad biological functions, including key roles in drug metabolism, breakdown of xenobiotics, and steroid and secondary metabolite biosynthesis [ 18]. More than 4,500 sequences of this ubiquitous enzyme are known [ 19]. Members of the artificial family of chimeric P450s reported here differ from any known protein by up to 109 amino acids, yet most retain significant catalytic activity. Unlike natural protein families, this artificial family also includes sequences that do not fold or function. Inclusion of nonfunctional sequences enables us to apply powerful logistic regression tools [ 20] to the multiple sequence alignment (MSA) of the laboratory-generated proteins and determine which elements contribute to correct heme incorporation and retention of catalytic activity in the cytochrome P450 heme domain.

## Results/Discussion

### SCHEMA Design and Construction of a Chimeric P450 Library

**Figure 1:**
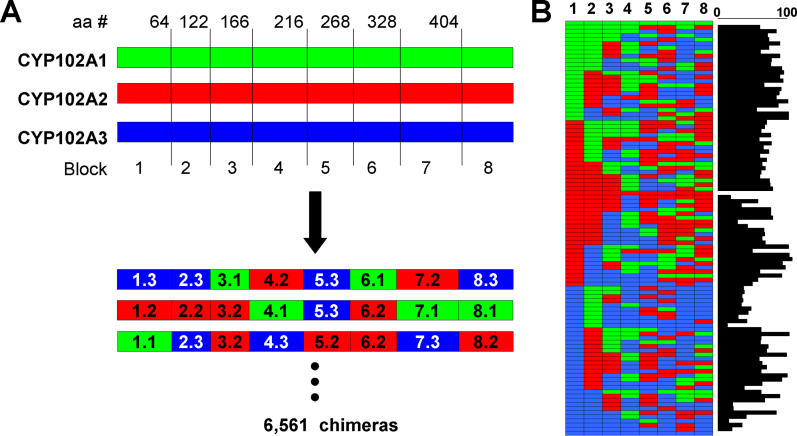
Diverse Chimeras Created by Site-Directed Recombination (A) Site-directed recombination of three bacterial cytochromes P450 showing crossover sites chosen to minimize the number of disrupted contacts (number is last residue of the sequence block according to CYP102A1 numbering). Blocks are assigned numbers 1 through 8 and three fragments are possible at each block. Three example chimeras are shown to illustrate the fragment nomenclature, e.g., fragment 1.3 is block 1 inherited from parent A3. (B) Sequences of three parents and 97 folded P450 chimeras and number of amino acid changes relative to the closest parent (bar on right).

We generated an artificial family of cytochromes P450 by recombining fragments of the genes encoding the heme-binding domains of three bacterial P450s, CYP102A1 (also known as P450 _BM3_), CYP102A2, and CYP102A3 (abbreviated A1, A2, and A3), which share ˜65% amino acid identity [ 21, 22] ( Figure 1). The parent proteins are 463–466 amino acids long and contain the single substitution F87A (A1) or F88A (A2 and A3), which increases the peroxygenase activities of these heme domains [ 23]. Calculations of the SCHEMA disruption that results when residue–residue contacts present in the parent structure are broken by recombination (see Materials and Methods) served to guide the placement of crossovers so as to maximize the number of highly mutated, folded proteins in the resulting library.

To accomplish this, we used the structure of the heme domain from CYP102A1 [ 24] to computationally evaluate 5,000 libraries with seven crossovers, each of which contained 3 ^8^ = 6,561 chimeric sequences (including the parents). Crossover sites were chosen randomly, with a minimum fragment size of 20 residues. To estimate the fraction of folded proteins in each library, we counted the number of structural contacts, E, disrupted in each chimeric sequence (see Materials and Methods) [ 14, 16]. Based on data from 17 A1–A2 chimeras individually constructed and studied previously [ 25], we modeled the probability of folding as a step function which decreases from 1 to 0 at a threshold of E = 30. Fraction folded was thus calculated as the number of chimeras in each library with E ≤ 30 divided by the total number of chimeras (= 6,561). The average number of amino acid substitutions from the closest parent <m> for the folded proteins (those with E ≤ 30) was also calculated as a measure of the library sequence diversity.

From the set of 5,000 randomly generated libraries, we selected only those with a fraction folded greater than 25% for further study. Within these, 14 crossover locations dominated, appearing in more than 40% of the libraries. Using these 14 crossover sites, we evaluated all 3,432 possible seven-crossover libraries and chose one with a high fraction folded (40%), high diversity (<m> = 68 for the chimeras with E ≤ 30, <m> = 76.4 for the library as a whole), and crossovers distributed over the primary sequence (average number of residues per block = 59 ± 10). The final design has crossovers located after residues Glu64, Ile122, Tyr166, Val216, Thr268, Ala328, and Gln404, based on the numbering of the A1 sequence ( Figure 1A).

**Figure 2:**
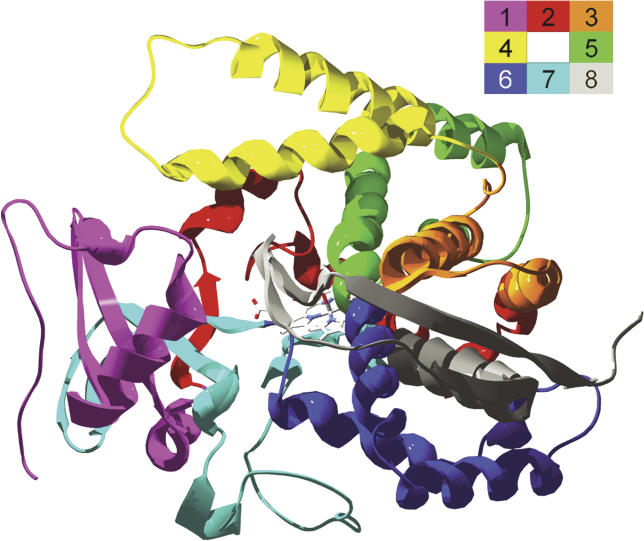
Structural Model of Heme-Domain Backbone Structure Showing Positions of Each Block Model is based on the crystal structure of CYP102A1 (2HPD) [ 26]. Blocks are color-coded as shown and heme is shown in CPK coloring.

**Figure 3:**
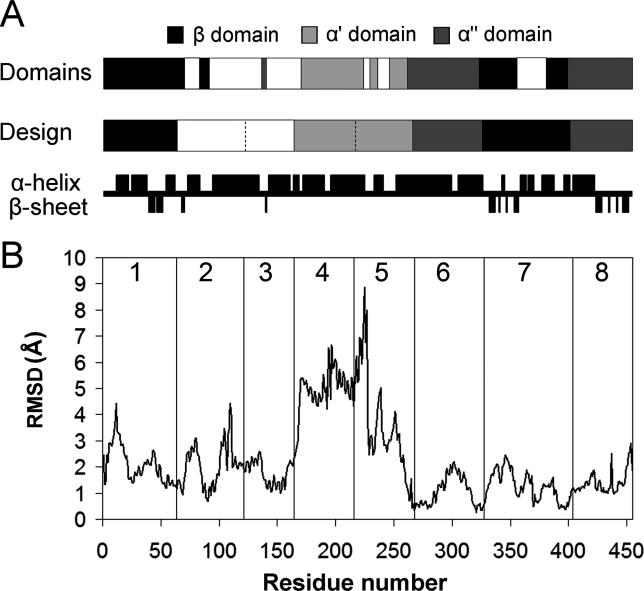
Comparison of Library Design to Domains, Dynamics, and Secondary Structure of CYP102A1 (A) Crossovers in the library designed using the SCHEMA energy function capture domain boundaries of CYP102A1 determined from molecular dynamics simulations [ 27]. Crossovers between blocks 2–3, 4–5, 5–6, and 7–8 lie within α-helices. (Secondary structure assignment is based on the CYP102A1 crystal structure [ 24]). (B) Plot of the RMSD between the backbone atoms of the substrate-bound (closed) and unbound (open) structures of CYP102A1. The RMSD was calculated by comparing molecule B of the substrate-free structure [ 29] and molecule A of the structure bound to palmitoleic acid [ 26] using Swiss PDB Viewer. Vertical lines designate crossover locations and blocks are numbered. Crossovers between blocks 1–2, 5–6, 6–7, and 7–8 occur at positions that move < 1.2 Å between the two structures. Crossover 3–4 is located next to a region of high identity and may be shifted towards the N-terminus by up to 14 residues and still produce the same chimeras. This shift allows it to occur at a position which moves < 1.2 Å.

The individual structural elements identified by SCHEMA are not obvious based on secondary or domain structure ( Figures 2 and 3A). For example, the crossovers between blocks 2–3, 4–5, 5–6, and 7–8 lie within the D, G, I and L helices, respectively [ 26]. Individual blocks, however, combine to form larger structural elements that coincide with protein domains determined from inspection of the A1 crystal structure [ 26] and concerted motions evident in molecular dynamics simulations of the same protein [ 27] ( Figure 3A). Blocks 1 and 7 comprise the independent “β domain,” most of which is a five-stranded β-sheet. The two-stranded, anti-parallel β-sheet comes from block 7, while the remaining three β-strands are contributed by block 1. The library design divided this domain into the fewest possible pieces. The remaining blocks comprise the “α domain” [ 26], which on the basis of concerted protein motions has been divided further into α′ (corresponding to blocks 4 and 5) and α″ domains (blocks 6 and 8) [ 27]. These three domains reflect groups of residues that move together not only in molecular dynamic simulations but also between different conformations of A1, which undergoes a large conformational change upon substrate binding [ 28]. Considering the root-mean-square deviation (RMSD) between the substrate-bound (closed) and substrate-free (open) forms of A1 ( Figure 3B) [ 29], five of seven crossovers are in regions which move 1.2 Å or less, significantly less than the average displacement of 2.2 Å, and capture the boundaries of the previously defined domains within six residues.

The three gene fragments encoding each of the eight blocks were combinatorially assembled using the sequence-independent site-directed chimeragenesis (SISDC) [ 30] method developed specifically for this application to generate a gene library containing 6,561 different sequences ( Figure 1A). These genes were expressed in *Escherichia coli,* where high-throughput sequencing by DNA probe hybridization and functional assays determined the sequences and functions of the proteins they encoded.

### Sequence Analysis

Because the crossover locations are fixed, the complete sequence of a chimera (absent any point mutations, insertions, or deletions) can be obtained by determining which parent sequence is present at each block by DNA probe hybridization [ 31]. Out of 1,512 randomly selected colonies analyzed this way, 754 complete sequences were obtained. Of these, 628 were unique. The distribution of fragments in this sample revealed two main biases from the ideal incorporation of 33% of each parent at each block ( Figure S1): at block 1, parent A1 is present in 10% of the chimeras, while parent A2 is present at block 4 in only 0.5%.

We completely sequenced 39 chimeras in order to assess the frequency of point mutations and of insertions, deletions, and remaining tag sequences (indels). Tag sequences were inserted at each crossover location for library construction by SISDC, and any remaining tag sequences result in a large insertion. In seven randomly chosen chimeras we found only one synonymous point mutation and no indels. We also sequenced 32 randomly chosen chimeras for which folding status had been determined. Twenty of these encoded folded P450s, while 12 encoded proteins that were not P450s. In the 20 folded P450 sequences, there were zero remaining tag indels and two point mutations. In the 12 not-folded sequences, one point mutation and one remaining tag sequence were found. From the overall point mutation frequency of 0.007% (in 51,568 nucleotides), we estimate that fewer than 10% of the chimeras in the library contain a point mutation. No indels or tag sequences were found in any of the folded P450 sequences, and fewer than 9% of the not folded chimeras contain indels or tags. Comparing the results from DNA sequencing and probe hybridization analysis, we found that probe hybridization identified the correct fragment at all eight blocks in 31 of 32 sequences. Thus the sequencing information from probe hybridization reflects the true sequences of the chimeras with errors in less than 10% of the chimeras, the majority of which are due to single point mutations.

### Assignment of Folding Status

Using high-throughput CO difference spectroscopy [ 32], we assayed clones from the chimeric P450 library for the characteristic Soret peak at 450 nm. The presence of this peak indicates correct heme binding and thus a properly folded P450 heme protein. Of the 628 unique full-length sequences, 293 (47%) encoded folded P450s. Additional sequencing of folded P450s yielded an expanded dataset containing 955 unique sequences (including the three parents), of which 620 correctly incorporate heme and 335 do not ( Table S1). Thirty-eight of these 335 not-folded sequences gave a peak at 420 nm, characteristic of improperly incorporated heme and a nonfunctional enzyme [ 33, 34]. The remaining not-folded sequences lack a compatible heme-binding site and likely do not fold into a well-defined structure.

**Figure 4:**
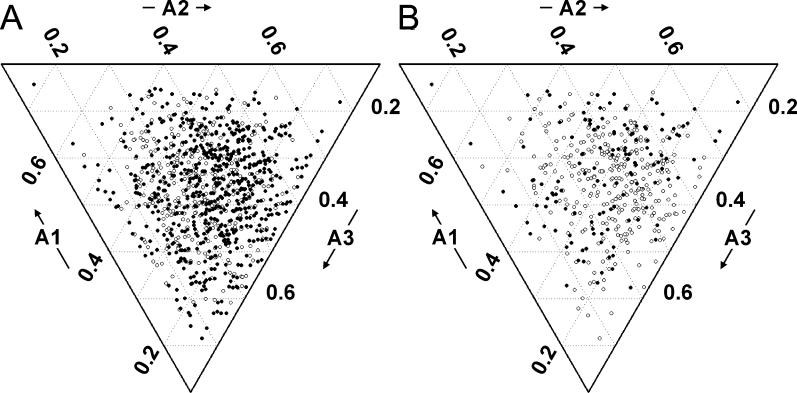
Ternary Diagrams Showing the Distribution of Chimera Amino Acid Compositions (A) Compositions of 955 folded (closed circles) and not-folded (open circles) chimeric sequences. Each data point represents the relative amino acid identity between a chimera and each parental sequence not including positions conserved between all three parents. This distance was calculated by determining the number of amino acids a chimera shares with each parent and dividing by their sum. The three relative identities add up to one. Since each parent shares some sequence identity with the other two, they do not lie at the corners of the diagram. (B) Compositions of 441 chimeras tested for activity on 12-pNCA: active chimeras (closed circles) and not active (open circles). Chimeras composed mostly of A3 and chimeras near the center tend to be inactive on 12-pNCA.

The folded sequences are highly mosaic and differ from their parents by 72.5 amino acids on average, with as many as 109 amino acid substitutions from the nearest parent sequence ( Figure 1B and Table S1). The average number of disruptions (<E>) is lower in chimeras that bind heme (29.5) versus those that do not (34.8). The average number of mutations in the heme-binding chimeras is also lower, 72.9 versus 77.5. The compositions of chimeras can be easily visualized using ternary diagrams ( Figure 4). For example, the sequence biases against single A1 and A2 fragments in the library construction generates fewer chimeras whose compositions are very close to A1 or A2 ( Figure 4A). It is clear from this plot, however, that the overall compositions of folded and not-folded chimeras are not markedly different and are well distributed over the accessible composition space.

### Catalytic Activities of Folded P450 Chimeras

**Figure 5:**
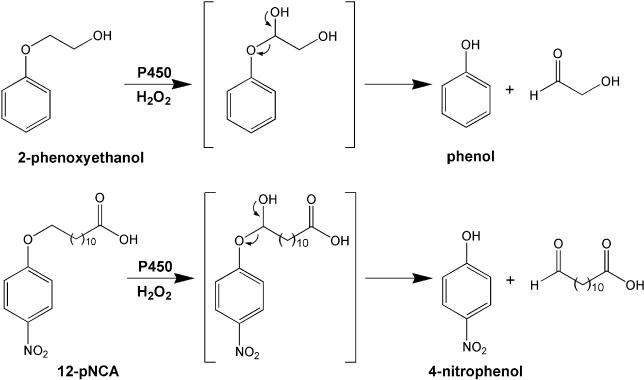
Substrates and Major Products of P450 Peroxygenase Reactions with 2-Phenoxyethanol and p-Nitrophenoxydodecanoic Acid (12-pNCA) In both cases, hydroxylation yields a hemiacetal which decomposes to phenolic products detectable in high-throughput assays.

We estimated the fraction of chimeras that are functional by assaying 320 folded P450 chimeras for peroxygenase activity on 2-phenoxyethanol, a substrate accepted by all three parents. Reaction on this substrate yields phenol ( Figure 5), which is detectable in high throughput [ 35]. The three parent P450s naturally occur as fusion proteins to an FAD- and FMN-containing NADP reductase [ 21]. These monooxygenases use NADPH and molecular oxygen to hydroxylate fatty acids [ 22]. The parent heme domains, by virtue of the single amino acid substitutions F87A in A1 and F88A in A2 and A3, also function as peroxygenases, catalyzing oxygen insertion in the presence of hydrogen peroxide [ 23, 25]. Chimeras that produced at least 25% of the total product formed in the assay by the most active parent (A1) were considered active. Of the 320 folded chimeras assayed, 72% were found to be active on 2-phenoxyethanol.

We also assayed all the 955 chimeras for which the sequences and folding status were determined for activity on the fatty acid analog p-nitrophenoxydodecanoic acid (12-pNCA, Figure 5). The parent A1 and A2 heme domains are active on 12-pNCA, while A3 is not. Chimeras with 25% of the total product formed by A1 during the assay were considered active. None of the chimeras that did not fold properly showed activity. We then determined activity status for folded P450s whose concentration was at least 500 nM, in order to remove false negatives based on low expression or other experimental errors. Of the folded chimeras, 441 met this constraint, of which 134 (30%) were active on 12-pNCA ( Table S1). The average number of disruptions is lower for chimeras active on 12-pNCA versus those that are not (<E> = 26.3 versus 31.4). Mutations are similarly lower in active chimeras (<m> = 70.9 versus 76.9).

A ternary diagram showing the 441 chimeras tested for activity on 12-pNCA ( Figure 4B) demonstrates that the sampled sequences are distributed similarly to the larger dataset ( Figure 4A). Parent A3 is inactive on 12-pNCA, and there are only a few chimeras with a high fraction of sequence from A3 that exhibit this activity. Additionally, there is a lower density of active chimeras near the center, where the chimeric sequences have the greatest divergence from the parents.

Fewer chimeras showed activity on 12-pNCA than on 2-phenoxyethanol, which we attribute to the fact that one parent, A3, is not active towards 12-pNCA, while all three parents are active on 2-phenoxyethanol. Overall, 73% of the folded chimeras assayed exhibited peroxygenase activity on at least one of these two substrates. Thus, at least 35% of the 6,561 sequences in the library are folded and functional, corresponding to 2,300 new P450 enzymes, not including any that are active on substrates not tested. This functional fraction is roughly three times higher than reported in a study in which more closely related cytochromes P450 (>71% amino acid identity) were recombined using a DNA shuffling methodology that leads to crossovers at regions of high sequence identity [ 36].

### Thermostabilities of Folded P450 Chimeras

To examine how recombination affects protein stability, we measured the melting temperatures of the parent P450s and 14 chimeras (all of which denature irreversibly at high temperature) by monitoring the disappearance of the P450 Soret peak with increasing temperature. A range of T _m_'s (42 °C–62 °C) was observed in this small sample ( Table 1). The most stable chimera differs from its closest parent by 84 amino acid substitutions, yet its melting temperature is 7 °C higher than the most stable parent. It is also higher than that of a variant of the A1 heme domain previously stabilized by sequential random mutagenesis and screening [ 37]. If a chimera is able to bind heme, then on average its stability appears not to be compromised relative to the parent proteins. The ability of the blocks to assemble into more thermostable proteins when removed from their natural context supports the modular nature of these elements and likely reflects some intrinsic stability of the individual blocks, due to the large number of structural contacts preserved by the library design.

**Table 1:**
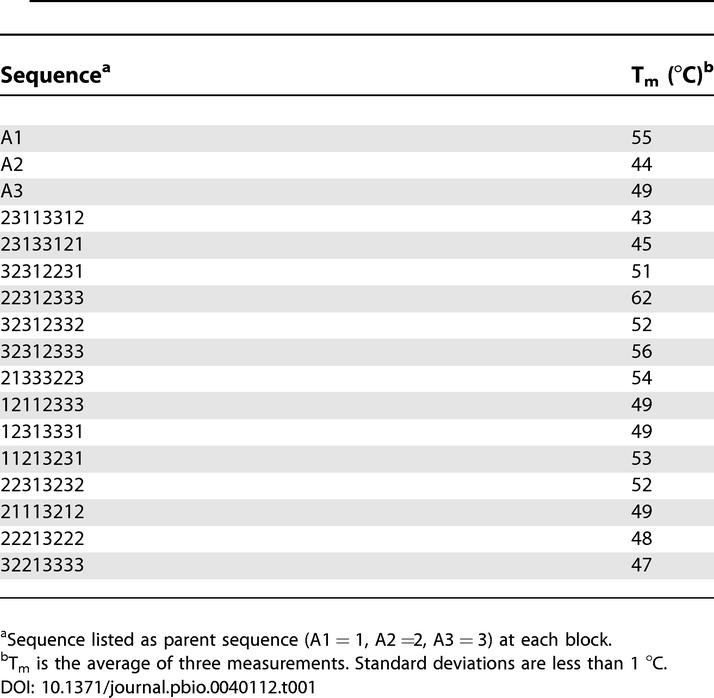
Thermostabilities of Parent and Chimeric Heme Domains

### Logistic Regression Analysis of the Multiple Sequence Alignments

Small sets of chimeric P450s have been constructed previously for investigations of sequence-structure-function relationships [ 38, 39]. The MSA of natural protein families are also widely used for this purpose. Comprised of sequences largely uncoupled from natural selection, including sequences that encode nonnatural functions (such as not folding or not functioning), the artificial protein family described here offers a unique opportunity to elucidate key sequence and structural contributions to P450 folding and function. By analyzing the MSAs of the chimeric P450s we can identify how different blocks and their parental identities influence folding and heme binding or catalytic activity. Because this dataset also includes sequences that encode not-folded and not-functional proteins, we can use logistic regression analysis (LRA), an analog of linear regression suitable for the type of binary data presented here, to analyze the MSAs. Other, more commonly used methods such as contingency table [ 40, 41] and statistical coupling [ 1, 42] are unable to utilize the additional information provided by the sequences that do not fold or function.

Underlying our LRA of the folded/not-folded dataset is the idea that individual fragments and interactions between fragment pairs contribute to whether a chimera will fold and bind heme. LRA fits an energy model containing intra- and inter-fragment terms; the magnitude of each term reflects how strongly that variable affects the likelihood of folding, with negative values increasing the likelihood and positive values decreasing it [ 20]. If energy is below a threshold, a chimera is assumed to be folded; otherwise it is not. In order to avoid overfitting the data, *p*-value testing is used to determine which fragments make a significant contribution to predicting chimera folding status.

**Figure 6:**
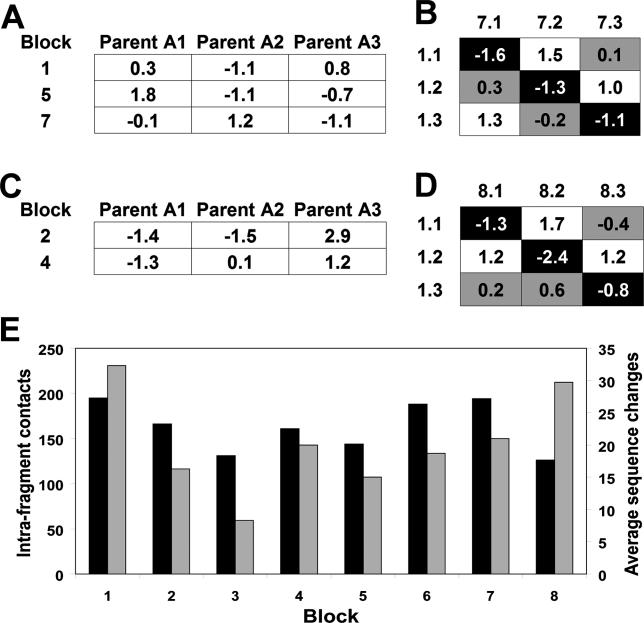
LRA of MSAs Identified Blocks and Block Pairs That Contribute to Whether a Chimera Folds and Binds Heme and Whether It Exhibits Activity on 12-pNCA (A) Intra-fragment terms in the energy model from LRA of folded/not-folded sequences indicate that blocks 1, 5, and 7 make significant contributions to folding and incorporation of heme. Negative energies increase the likelihood of folding and correctly binding heme while positive ones decrease it. (B) The single significant inter-fragment interaction from LRA of folded/not-folded sequences comes from pair 1–7 and includes the nine energy terms for pair 1–7, which can be divided into three groups. The on-diagonal elements (filled black) are the most stabilizing. The three terms filled gray have roughly average energy. The three white elements are destabilizing relative to the others. (C) Significant intra-fragment terms from LRA of the MSA of active/not-active sequences indicate that blocks 2 and 4 have significant effects on peroxygenase activity. (D) The single significant inter-fragment interaction between blocks 1 and 8, showing the nine terms, divided into similar groups as in part B. (E) Black bars, intra-fragment contacts within each block, as defined by the SCHEMA distance of 4.5 Å [ 16]. Gray bars, the average number of sequence changes between each parent.

We applied LRA to the MSA of the entire set of 955 chimeric P450s in Table S1 to determine which blocks contribute to folding and correct heme binding. The resulting energy model includes blocks and block pairs that are significant with the likelihood ratio test and cross-validation (see Materials and Methods). This analysis revealed that blocks 1, 5, and 7 by themselves and the interaction between blocks 1 and 7 (abbreviated 1–7) contribute significantly to whether a chimeric P450 folds and binds heme ( Figure 6). All other blocks and block pairs are apparently to a large extent interchangeable with respect to whether a chimera folds properly.

**Figure 7:**
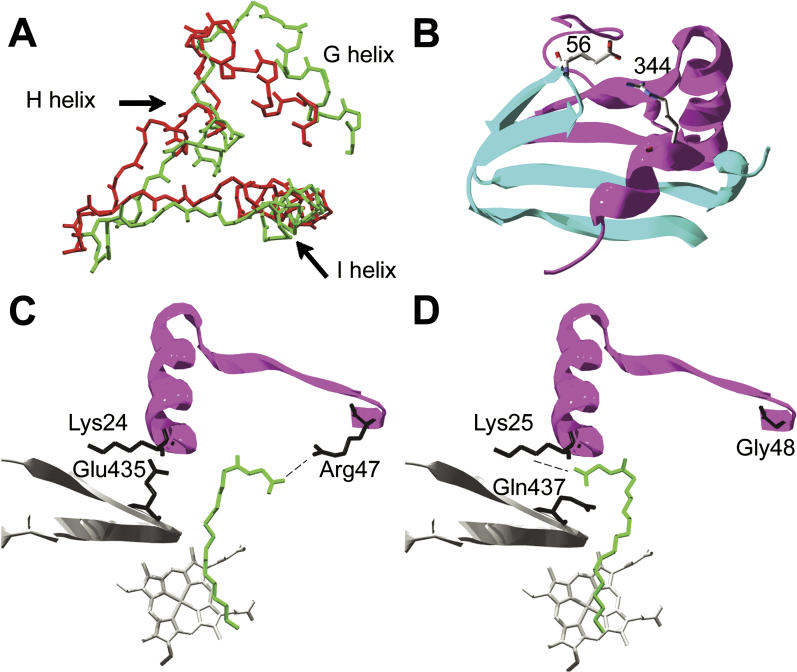
Structural Elements That Contribute Significantly to Proper Folding and Incorporation of Heme and Model of Substrate Binding in CYP102A1 and CYP102A2 (A) Movement of block 5 between open (red) and closed (green) structural forms based on alignment of heme cofactor. The average displacement over the whole block is 3.6 Å. (B) Residues that could contribute to positively and negatively interacting fragments at blocks 1 and 7. Residue 56 (shown as arginine) is an arginine, glutamate, and glutamine; and residue 344 (shown as glutamate) is a glutamate, lysine, and glutamate in A1, A2, and A3, respectively. The fragment pairs that result in unfavorable charge–charge interactions for these closely spaced side chains are unfavorable overall for folding and heme incorporation. (C) In CYP102A1 the carboxylate group of the fatty acid substrate (in green) interacts with arginine 47 from block 1 (dashed line). Residue 435, from block 8, and residue 24 may form a salt bridge. Portions of blocks 1 and 8 are shown in purple and grey, respectively. (D) Proposed model for CYP102A2 showing an alternative binding configuration for the fatty acid substrate. Residue 437 (in block 8) is a glutamine in A2. Thus in A2, lysine 25 is free to interact with the substrate carboxylate group (dashed line). Structure shown is 1FAG [ 29]. Amino acid residues are in black and heme is grey.

As shown in Figure 6A, the intra-fragment terms for fragments 1.2 and 7.3 have lower energy relative to the other parents, which means the sequence changes in these fragments are more favorable for heme binding. Blocks 1 and 7 are in fact expected to be important, because they contain the most residues, the greatest number of intra-fragment contacts ( Figure 6E), and block 1 has the highest average number of sequence changes, whereas block 7 has the third most ( Figure 6E). In contrast, block 5 has the third fewest intra-fragment contacts and the second fewest average number of sequence changes ( Figure 6E). At this block, fragment 5.1 is the least favored of all the fragments for folding and heme binding ( Figure 6A). Parent A1 contains a deletion relative to A2 and A3 in block 5, which may contribute to this behavior. We suspect that some of the importance of block 5 is due to the dynamic nature of cytochromes P450, similar to what has been observed in multiple sequence analyses of other protein families [ 2]. The F, G, and H helices (in blocks 4 and 5) undergo displacements of more than 5 Å between the substrate-bound and substrate-free forms of A1 [ 29], and block 5 moves an average of 3.6 Å ( Figure 7A). This portion of the enzyme acts as a “hinge” by which the F and G helices close down upon the substrate. Because none of the residues in block 5 that contact the heme differ among the three parents, the importance must stem from how variable amino acids in block 5 affect dynamics or interact with conserved residues.

Block pair 1–7 was the only pair revealed by LRA as significant for folding and incorporation of heme. Blocks 1 and 7 interact extensively to form the β-domain ( Figure 7B) and experience the largest average number of broken contacts when the blocks are inherited from different parents. As expected, chimeras that inherit blocks 1 and 7 from the same parent are more likely to fold and bind heme ( Figure 6B). This result supports the core hypothesis of SCHEMA and other penalizing energy functions [ 43] which assign the best possible score to these wild-type interactions.

Inspection of the sequences of the parents in these two blocks revealed an electrostatic interaction that could contribute to the pattern of energies in Figure 6B. Residues 56 (block 1) and 344 (block 7) are 2.8 Å apart in the A1 crystal structure ( Figure 7B). At position 56, parent A1 contains a positively charged arginine, A2 has a negatively charged glutamate, and A3 has a neutral glutamine. Residue 344 is a glutamate in A1 and A3, but lysine in A2. Thus the interaction 1.1–7.2 pairs arginine and lysine, while 1.2–7.3 pairs glutamate and glutamate, both of which are repulsive.

We repeated the logistic regression analysis to determine which blocks affect activity on 12-pNCA, independent of heme binding, by applying LRA to the subset of 441 folded chimeras for which presence or absence of activity on 12-pNCA had been determined ( Table S1). This analysis revealed that blocks 2 and 4 by themselves and block pair 1–8 contribute to whether a folded chimera is catalytically active on this substrate. At blocks 2 and 4, the fragments derived from parent A3 are detrimental to activity ( Figure 6C). These sequence elements likely account for A3′s lack of activity on this substrate, since sequence from this parent at other blocks has little affect on 12-pNCA activity in the chimeras. The importance of block pair 1–8 may reflect a difference between A1 and A2 with respect to substrate binding: when A1 or A2 is present at either block 1 or 8, activity is strongly dependent on whether the other block comes from the same parent ( Figure 6D). This indicates that there are one or more interactions between blocks 1 and 8 that must be preserved in order for the enzyme to be active on 12-pNCA.

### Residues Contributing to Peroxygenase Activity on 12-pNCA

We sought to determine what interactions(s) might be responsible for the importance of the 1–8 pair, using the sequence differences in parents A1 and A2 for guidance. One obvious difference occurs at the position corresponding to Arg47 in fragment 1.1, which is located at the opening of the active site and is thought to interact with the carboxylate group of fatty acid substrates [ 29]. Substitutions of this residue in the A1 holoenzyme significantly reduce catalytic activity [ 44, 45]. In A2, the equivalent residue is Gly48, a residue that favors the binding of polycyclic aromatic hydrocarbons when present in the A1 holoenzyme [ 46]. We tested the importance of R47 to peroxygenase activity by swapping the residues at position 47/48 in A1 and A2, i.e., making the single mutation R47G in A1 and G48R in A2. The R47G mutation in A1 reduced the initial rate nearly 25 fold (from 65.9 ± 8.5 to 2.7 ± 0.5 nmol product/nmol P450/min), making it comparable to the activity of A2. On the other hand, the G48R mutation in A2 had no effect on rate. This suggested to us that G48 in A2 does not interact with the substrate carboxylate group, as the equivalent residue appears to do in A1.

We postulated that the different mode of substrate binding could be facilitated by a positively charged residue elsewhere in the A2 sequence. Only a small portion of block 8, consisting of halves of two β-strands (residues 434 to 439), is located near the active site ( Figure 7C). Examination of the parental sequence alignment in this region ( Table S2), however, revealed no lysines or arginines unique to fragment 8.2. Because fragments 8.1 and 8.3 are equally incompatible with 1.2 according to the LRA, we looked for a residue between 434 and 439 that was shared by A1 and A3 but not A2. Residue 435 in A1 (437 in A2 and A3), which is a glutamate in A1 and A3 and a glutamine in A2, met these criteria.

We then swapped these residues by making the E435Q mutation in A1 and the Q437E mutation in A2. The E435Q mutation in A1 reduced catalytic rate by 8 fold, whereas the Q437E mutation completely abolished the activity of A2 ( Table 2). Having shown this residue to be important to activity in both parents, we next chose eight inactive chimeras containing unfavorable 1–8 block combinations to determine whether swapping these positions could “rescue” the activity. We introduced the Q437E mutation into four chimeras with fragments 1.1 and 8.2 and the E435Q mutation into four with fragments 1.2 and 8.1 ( Table 2). This single substitution was able to confer activity in two of the eight chimeras.

**Table 2:**
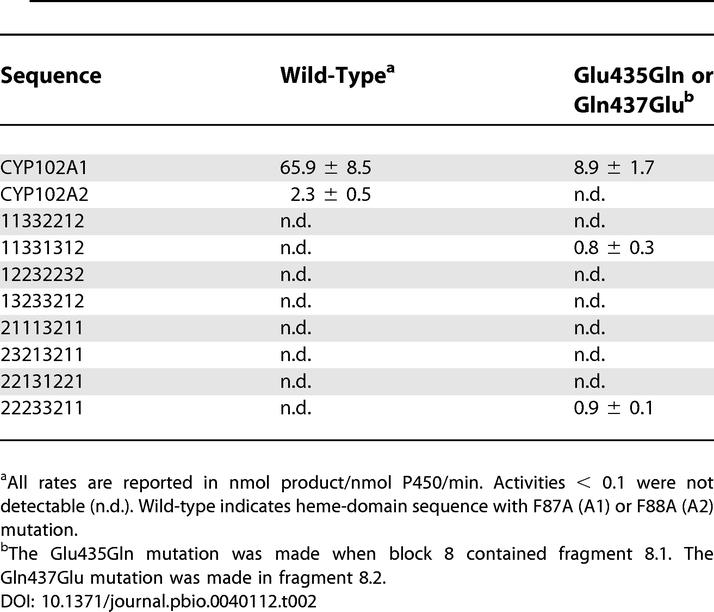
Peroxygenase Activities of Site-Directed Mutants of Parents CYP102A1 and CYP102A2 and Selected Chimeric Heme Domains on 12-pNCA

Thus the LRA analysis in combination with mutation studies uncovered a residue (Glu435/Gln437) previously unknown to be important for catalytic activity and suggests a different substrate binding mode in CYP102A2. One structural explanation for these results is illustrated in Figure 7C and 7D. Since A2 lacks a positive charge at position 48 and has no unique positively charged residues in the small portion of block 8 near the active site (or block 8 altogether), we hypothesized that another sequence change may have caused a positively charged residue to be made available elsewhere. Glu435 in A1 appears to participate in a salt bridge with Lys24, which is roughly 4 Å away in the crystal structure. The equivalent residue 25 is a lysine in A2 and a glutamine in A3. The lack of a salt bridge partner near Lys25 in A2 could free Lys25 to interact with the carboxylate tail of the fatty acid ( Figure 7D). In support of this, a single substitution of Gln437 to Glu rescued the activity of a chimera containing A2 sequence at block 8, but A1 sequence at block 1. Conversely, switching Glu435 to Gln in a chimera containing A1 sequence at block 8 but A2 sequence at block 1 was also able to rescue the activity. Of course, this single switch was unable to rescue activity in six more folded, but inactive chimeras, which indicates that additional interactions are also important (such as the contributions from residues in blocks 2 and 4).

### SCHEMA-Guided Recombination Creates a Library Rich in Properly Folded, Highly Mutated Sequences

The approach used here to identify optimal recombination sites differs from the SCHEMA profile described previously [ 14]. Evaluating libraries with randomly sampled crossovers, as was done here, and a recently developed global optimization of recombination sites [ 47] are both preferred over the SCHEMA profile, which neglects important structural interactions between amino acids distant in the primary sequence. Based on this design, three cytochromes P450 were divided into “building blocks” and combinatorially reassembled to yield a library in which 47% of the members fold and correctly bind heme. This folded fraction is slightly larger than the prediction of 40% from the design. The full library therefore contains an estimated 3,000 unique chimeric P450s, many of which are highly mutated compared to the parent P450s.

It is interesting to estimate the extent to which SCHEMA recombination has enriched the library relative to a library having the same distribution of mutation levels, but made using random mutagenesis. The fraction of folded proteins in a random library can be estimated using the protein's “neutrality,” or probability that a random amino acid substitution will not disrupt folding. Neutrality *ν* has been calculated for other proteins and ranges from 0.38 to 0.56 [ 7]. Using 0.6 as a conservative estimate for P450 neutrality, the fraction of folded P450s having a mutation distribution equaling that of the chimeras *(ff _r_)* is given by



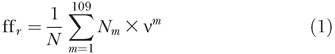



where *ν* = 0.60, *N* = total number of mutants (628, equal to the unique set of randomly sampled chimeras), *m* = number of amino acid changes, and *N _m_* = number of mutants with a given value of *m*. This yields a fraction folded *ff _r_* = 6.3 × 10 ^−5^. The fraction of folded chimeras in the library is 0.47, giving an enrichment of 0.47/ff _r_ = 7.5 × 10 ^3^. Thus, by this conservative estimate, SCHEMA-guided recombination has increased the frequency of folded chimeras by nearly four orders of magnitude.

### Conclusions

Protein families generated in the laboratory can be used to identify regions of the sequence and structure that are important for folding and function. This approach may be especially valuable for proteins with few naturally occurring family members. Datasets such as this one, containing hundreds of proteins for which functional information can be determined in high-throughput assays, will be invaluable for developing and validating structure prediction tools and for protein sequence-structure-function analysis. Finally, rich in sequence diversity as well as the ability to fold properly, these proteins may be sources of novel functions for laboratory protein evolution.

## Materials and Methods

### Calculation of SCHEMA disruption

The parent heme-domain sequences of A1, A2, and A3 were aligned using ClustalW [ 48] ( Table S2). The number of broken contacts in a chimera E [ 14, 16] is



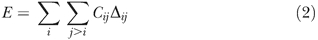



where the *C _ij_* are elements of the contact matrix which depend solely on the protein structure. Specifically, *C _ij_* = 1 if residues *i* and *j* are within 4.5 Å in the structure of A1 bound to N-palmitoylglycine (1JPZ) [ 24]; otherwise *C _ij_* = 0. The SCHEMA delta function Δ *_ij_* uses only the parental sequence alignment: Δ *_ij_* = 0 if the amino acids found in the chimera at positions *i* and *j* are also found together in any single parent at the same positions. Otherwise, the *i*– *j* contact is considered broken, and Δ *_ij_* = 1.

### Library construction

The heme domains of A1 and A2 were described previously [ 25]. The heme domain (first 1,401 nucleotides) of the A3 gene (a gift from Claes von Wachenfeldt, Lund University) was subcloned into the BamHI/EcoRI sites of the pCWori expression vector [ 49], and the mutation corresponding to F88A was introduced. The chimeric library was constructed following the SISDC method [ 30], using the type IIb restriction endonuclease *Bsa*XI. The full-length library was ligated into the pCWori vector and transformed into the catalase-deficient E. coli strain SN0037 [ 50]. Additional details can be found in Protocol S1.

### Probe hybridization analysis

Probe hybridization was performed as described [ 31] and detailed in Protocol S1.

### High-throughput carbon monoxide binding assay

Clones grown in 96-well plates were replicated into 500 μl of Luria-Bertani (LB) medium with 100 μg/ml ampicillin in 2 ml deep-well plates (BD Falcon, San Jose, CA) and grown in a humidified shaker (Kuhner ISF-1-W, Birsfelden, Sweden) for 20 h at 210 rpm, 30 °C and 80% relative humidity. Samples (150 μl) of these saturated cultures were transferred to 850 μl of terrific broth (TB) medium supplemented with 117 μg/ml ampicillin, 30 μg/ml thiamine, 0.6 mM δ-aminolevulinic acid, and 0.7 mM IPTG. These were grown for 24 h at 210 rpm, 25 °C and 80% relative humidity and harvested by centrifugation at 4 °C, 4,900 × g. Cell pellets were stored frozen at −20 °C until they were resuspended in 300 μl of lysis buffer (100 mM Tris [pH 8.2] with 0.5 mg/ml lysozyme and 2 units/ml DNAse) using a pipetting robot (Beckman Multimek 96, Fullerton, CA). Plates were incubated at room temperature for 1 h, followed by centrifugation at 4,900 × g for 10 min at 4 °C to clear the lysate. CO binding assays were carried out as described [ 32] and detailed in Protocol S1.

### Functional assays

Chimeras were assayed for peroxygenase activity on 12-pNCA in 96-well plate format as described [ 51]. Reactions were carried out in a volume of 200 μl with 250 μM 12-pNCA and 20 mM H _2_O _2_ in 100 mM Tris (pH 8.2) at room temperature and monitored at 410 nm for 30 min for accumulation of 4-nitrophenol. Chimeras in wells with total product formation greater than 25% of the average of four control wells with the A1 heme domain after 30 min were considered active (corresponding to > 5 μM product).

Activity on 2-phenoxyethanol was assayed in 96-well plates using the 4-aminoantipyrine assay (4-AAP), which detects phenol-like compounds [ 35]. Reactions were carried out in 120 μl with 1% DMSO, 1% acetone, 100 mM 2-phenoxyethanol and 20 mM H _2_O _2_ in 100 mM N-[2-hydroxyethyl]piperazine-N′-[3-propanesulfonic acid] (EPPS) [pH 8.2]. Reactions were mixed and left at room temperature without shaking for 2 h then quenched with 120 μl of 0.1 M NaOH and 4 M urea. Thirty-six μl of 0.6% 4-AAP was added, the 96-well plate reader was zeroed at 500 nm, and 36 μl of 0.6% potassium persulfate was added. After 20 min the A _500_ was read. Chimeras in wells with an A _500_ greater than 25% of the average of four control wells with the A1 heme domain were considered active, corresponding to > 20 μM product.

### Thermostability

Thermostabilities (as described by T _m_, the temperature at which half of the protein is unfolded) were measured using CO difference spectroscopy to monitor the disappearance of the Soret band with increasing temperature as described [ 25].

### Logistic regression analysis

The significance of each block (intra-fragment) and block pair (inter-fragment) was calculated relative to a reference model with all eight blocks using the likelihood ratio test [ 20]. In the case of heme binding, this identified six potentially significant variables which were collected into a second-round reference model and reevaluated using the likelihood ratio test ( Table S3). Blocks 1, 5, 7, and block pair 1–7 remained highly significant in the second round, whereas pairs 1–5 and 5–8 dropped in significance to *p* > 10 ^−3^, a threshold established previously [ 20]. Cross-validation tests (data not shown) provide further evidence that only the variables 1, 5, 7, and 1–7 are significant. The same analysis was done for activity on 12-pNCA and determined blocks 2, 4 and 1–8 are significant.

### Construction and analysis of site-directed mutants

Single mutations were made in the A1 and A2 genes and in the genes of the eight chimeras seen in Table 2. The R47G and G48R mutations were made using the codon from the alternate parent, Arg (CGT) and Gly (GGC), respectively. The E435Q and Q437E mutations were made in the same fashion with the codons Glu (GAA) and Gln (CAA) being swapped. Mutants were constructed using PCR overlap extension mutagenesis [ 52], cloned into the BamHI/EcoRI site of pCWori and transformed into catalase-deficient *E. coli.* P450 chimeras and parents were cultured in 200 ml of TB medium and the initial rates on 12-pNCA were measured with 1 μM enzyme, 250 μM 12-pNCA, 1% DMSO, 20 mM H _2_O _2_ in 100 mM Epps (pH 8.2), as done previously [ 25].

## Supporting Information

Figure S1 Fragment Distribution at Each Block Based on Probe Hybridization of Genes from 754 Unselected Clones (37 KB PDF)

Protocol S1 Experimental Procedures: Library Construction, Probe Hybridization Analysis, and High-Throughput Carbon Monoxide Binding Assay (74 KB PDF)

Table S1 955 Chimeric and Parent P450 Heme Domain Sequences with their Folding State, 12-pNCA Activity State, and Number of Sequence Changes (25 KB PDF)

Table S2 ClustalW Amino Acid Sequence Alignment of the Heme Domain of CYP102A1 (Residues 1–463), CYP102A2, and CYP102A3 (Residues 1–466) Numbering is according to the CYP102A1 sequence.
(11 KB PDF)

Table S3 Significance of the Top Six Variables Identified by Logistic Regression Analysis of Cytochrome P450 Chimeras (10 KB PDF)

Table S4 Primers Used for Site-Directed Recombination of Cytochromes P450 Heme Domains of A1, A2, and A3 (26 KB PDF)

Table S5 Sequences of Probes for Hybridization Analysis (19 KB PDF)

### Accession Numbers

The NCBI Entrez ( http://www.ncbi.nlm.nih.gov/gquery/gquery.fcgi) accession numbers for the genes and gene products discussed in this paper are CYP102A1 (J04832), CYP102A2 (CAB12544) and CYP102A3 (U93874).

## Abbreviations

12-pNCA: p-nitrophenoxydodecanoic acid
A1: CYP102A1
A2: CYP102A2
A3: CYP102A3
LRA: logistic regression analysis
RMSD: root-mean-square deviation
SISDC: sequence-independent site-directed chimeragenesis
