# Supplementary material for: Structure-Guided Recombination Creates an Artificial Family of Cytochromes P450
Source: PLoS Biol. 2006 Apr 11;4(5):e112. doi: 10.1371/journal.pbio.0040112 (PMC1431580; doi:10.1371/journal.pbio.0040112)
Supplement: Supplementary file 1 [file pbio.0040112.sd001.pdf]

Site-Directed Recombination Creates an Artificial Family of Cytochromes P450

Christopher R. Otey, Marco Landwehr, Jeffrey B. Endelman, Kaori Hiraga, Jesse D. Bloom and Frances H. Arnold

Experimental Methods

Library construction. The chimeric library was constructed following the sequence-independent site-directed chimeragenesis (SISDC) method [1], using the type IIb restriction endonuclease *Bsa*XI. This required removal of a *Bsa*XI site in parent A1 using a synonymous mutation at Gly368 (GGA to GGT). N- (blocks 1-4) and C-terminal (blocks 5-8) half-length parental genes were constructed separately with a tag sequence containing a *Bsa*XI recognition site at each of the three crossover locations (DNA primers used for construction are listed in Table S4). The half-length genes were cloned into the pCR®-Blunt II-TOPO plasmid (Invitrogen) and their DNA sequences were confirmed by sequencing (Laragen, Los Angeles, CA). Digestion of the half-length parental genes with *BsaX*I to remove the tag sequences yielded DNA fragments with unique 3 bp overhangs at each crossover site. After column purification to remove the small (30 bp) tags, the fragments from three parents were mixed in equimolar amounts and ligated to make two half-length chimeric libraries. Digestion with *Nhe*I (restriction site designed into tag sequence) and *BsaXI* after ligation removed genes with residual tags from the final library. Each half-length library was PCR amplified, cloned back into the TOPO vector and transformed into E*scherichia coli* TOP10 cells (Invitrogen).

The N- and C-terminal half-libraries were digested with *Bgl*II/*Sal*I, and *Sal*I/*Mfe*I, respectively, and cloned into the *BamH*I/*EcoR*I sites of the pCWori vector. The *Sal*I recognition site corresponds to crossover site 216-217 and was introduced into all three P450 genes synonymously. The N- and C- terminal libraries were ligated at this site to yield the full-length library, which was transformed into the catalase-deficient *E. coli* strain SN0037 [2].

Probe hybridization analysis. Probe hybridization was performed as described [3,4]. Twenty-four unique oligonucleotide probes were designed to specifically recognize each parent fragment with a T_m ~63°C (Table S5). Probes were labeled using the terminal transferase reaction with either fluorescein (Amersham) or digoxigenin (Roche) following the manufacturer's protocol. Anti-fluorescein (Amersham) or anti-digoxigenin (Roche) antibodies conjugated to alkaline phosphatase were used for chemiluminescent detection according to the manufacturer's protocol and recorded with a Gel-Doc system from BIORAD or BIOMAX films from Kodak.

High-throughput carbon monoxide binding assay. CO binding assays were carried out as described [5] with minor modification. Lysate (160 µl) was added to wells of a 96-well plate followed by addition of 40 µl of freshly-prepared 0.1 M sodium hydrosulfite (in 1.3 M phosphate buffer, pH 8.0). Using a Spectra Max Plus 384 plate reader (Molecular

Devices), blank spectra were taken every 10 nm from 400 to 500 nm, as were readings for 450 and 490 nm. Plates were put in a vacuum oven and the atmosphere was removed (to ~350 mbar) and replaced with CO. Plates were allowed to incubate for 5 to 10 minutes, at which point the spectra and absorbencies at 450 and 490 nm were read. Assays were performed in duplicate.

Chimeras were assigned a folding status (folded P450 = 1, not folded = 0) based on the following. To be folded, the spectrum must have a distinct Soret peak within an absorbance range of -0.01 to 0.01 at 450 nm. In addition, the minimum CO difference value ($A_{450} – A_{490}$) must be ≥ 0.0015. The background, as assayed using cells with a null vector (pCWori with no insert), was $4.9 \times 10^{-4} \pm 4.2 \times 10^{-4}$, which set 1.5×10^{-3} as the minimum CO difference value giving greater than 95% confidence (greater than two standard deviations). If the sequence was not assigned folded status – 1, it was deemed 'not folded' = 0.

1. Hiraga K, Arnold FH (2003) General method for sequence-independent site-directed chimeragenesis. J Mol Biol 330: 287-296.

2. Nakagawa S, Ishino S, Teshiba S (1996) Construction of catalase deficient Escherichia coli strains for the production of uricase. Biosci Biotechnol Biochem 60: 415-420.

3. Meinhold P, Joern JM, Silberg JJ (2003) Analysis of shuffled libraries by oligonucleotide probe hybridization. Methods Mol Biol 231: 177-187.

4. Joern JM, Meinhold P, Arnold FH (2002) Analysis of shuffled gene libraries. J Mol Biol 316: 643-656.

5. Otey CR (2003) High-throughput carbon monoxide binding assay for cytochromes P450. Methods Mol Biol 230: 137-139.
