# Supplementary material for: Structure-Guided Recombination Creates an Artificial Family of Cytochromes P450
Source: PLoS Biol. 2006 Apr 11;4(5):e112. doi: 10.1371/journal.pbio.0040112 (PMC1431580; doi:10.1371/journal.pbio.0040112)
Supplement: Supplementary file 2 [file pbio.0040112.sg001.pdf]

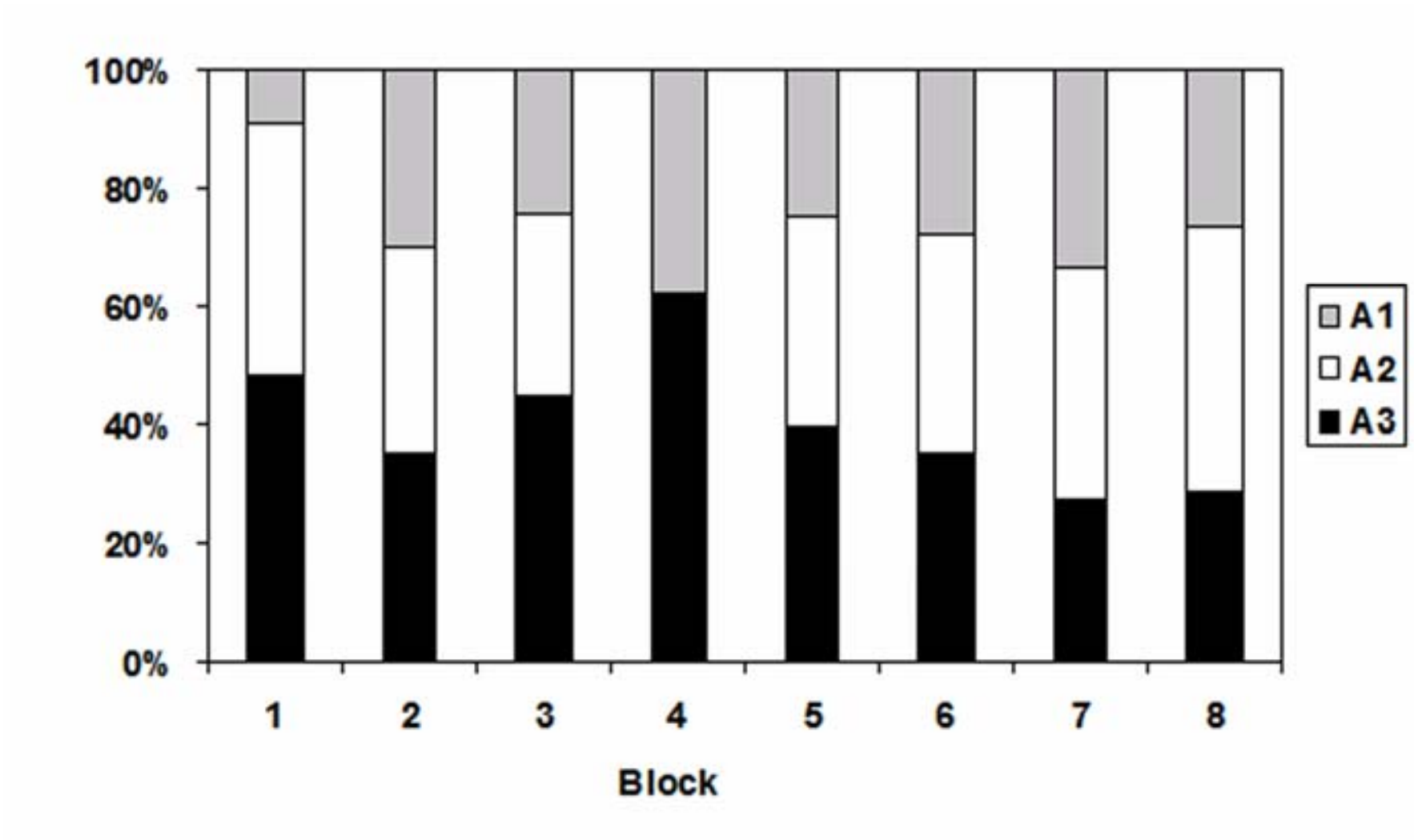

Figure S1. Fragment distribution at each block based on probe hybridization of genes from 754 unselected clones.
