# Supplementary material for: Structure-Guided Recombination Creates an Artificial Family of Cytochromes P450
Source: PLoS Biol. 2006 Apr 11;4(5):e112. doi: 10.1371/journal.pbio.0040112 (PMC1431580; doi:10.1371/journal.pbio.0040112)
Supplement: Supplementary file 3 [file pbio.0040112.st001.pdf]

Table S1. 955 chimeric and parent P450 heme domain sequences with their folding state, 12-pNCA activity state and number of sequence changes.

	Folding State[2]	12-pNCA activity[3]	m[4]
11111111	1	1	0
11112212	1	1	62
11113233	1	--	86
11113311	1	1	36
11131313	1	--	73
11133231	1	--	75
11212112	1	1	51
11212333	1	1	91
11213133	1	--	75
11213231	1	1	63
11232111	1	--	44
11232232	1	--	91
11232333	1	--	70
11311233	1	1	79
11312233	1	1	93
11313233	1	1	85
11313333	1	1	69
11331312	1	0	78
11331333	1	0	64
11332212	1	0	92
11332233	1	0	79
11332333	1	--	63
11333212	1	0	94
12112333	1	1	97
12113221	1	1	77
12211232	1	1	91
12211333	1	1	96
12212112	1	--	68
12212211	1	1	60
12212212	1	1	80
12212332	1	1	93
12213212	1	1	90
12232111	1	1	61
12232112	1	--	89
12232232	1	0	74
12232233	1	0	90
12232332	1	0	90
12233212	1	0	92
12313331	1	1	80
12322333	1	1	86
12331123	1	--	103
12331333	1	1	68
12332223	1	--	89
12332233	1	1	83
12332333	1	--	67

Sequence[1]	Folding State[2]	12-pNCA activity[3]	m[4]
31233233	1	--	37
31233333	1	--	21
31311231	1	1	81
31311233	1	0	67
31311332	1	1	80
31312113	1	1	88
31312133	1	1	70
31312212	1	0	100
31312222	1	--	76
31312231	1	--	95
31312233	1	1	66
31312323	1	1	71
31312332	1	1	79
31312333	1	1	50
31313111	1	--	59
31313131	1	1	77
31313132	1	1	84
31313133	1	--	55
31313223	1	--	72
31313232	1	1	80
31313233	1	--	51
31313333	1	1	35
31331331	1	--	62
31331333	1	0	30
31332131	1	0	81
31332133	1	--	49
31332232	1	0	74
31332233	1	0	45
31332312	1	--	76
31332322	1	--	79
31332323	1	--	50
31332333	1	--	29
31333233	1	0	30
31333322	1	--	64
31333332	1	0	43
31333333	1	0	14
32111333	1	1	64
32112212	1	--	85
32112321	1	--	105
32113131	1	0	85
32113232	1	1	93
32113233	1	1	64
32131133	1	--	63
32132232	1	0	79
32132233	1	--	58

12333331	1	--	84	32132331	1	0	74
12333333	1	1	52	32133111	1	--	88
13113311	1	0	50	32133232	1	--	72
13213131	1	--	57	32133233	1	--	43
13222212	1	--	76	32133331	1	--	59
13233212	1	0	104	32211323	1	0	83
13332333	1	0	49	32212133	1	0	81
13333122	1	--	104	32212231	1	0	101
13333131	1	--	78	32212232	1	0	73
13333211	1	0	80	32212233	1	1	77
21111321	1	0	78	32212321	1	0	96
21111323	1	0	107	32212323	1	1	82
21111333	1	0	90	32212332	1	0	89
21112122	1	1	68	32212333	1	1	61
21112123	1	0	97	32213123	1	--	87
21112132	1	1	89	32213132	1	--	95
21112212	1	1	72	32213231	1	0	94
21112222	1	1	48	32213333	1	1	46
21112232	1	1	69	32232131	1	--	92
21112311	1	0	68	32232322	1	--	65
21112312	1	1	88	32232331	1	--	72
21112322	1	1	64	32232333	1	--	40
21112332	1	1	85	32233222	1	--	64
21113111	1	0	50	32233332	1	--	54
21113112	1	1	78	32311131	1	--	78
21113122	1	1	83	32311323	1	1	76
21113133	1	1	94	32312212	1	--	83
21113211	1	0	70	32312231	1	1	102
21113212	1	1	87	32312233	1	--	70
21113221	1	--	91	32312311	1	--	94
21113223	1	0	92	32312322	1	1	75
21113312	1	1	98	32312323	1	--	75
21113322	1	1	79	32312331	1	--	86
21131121	1	0	79	32312332	1	--	83
21132112	1	1	89	32312333	1	1	54
21132113	1	0	101	32313133	1	--	59
21132212	1	1	69	32313231	1	0	87
21132222	1	0	45	32313232	1	0	84
21132311	1	0	89	32313233	1	1	55
21132313	1	--	86	32313313	1	--	57
21132321	1	0	89	32313332	1	--	68
21132323	1	0	89	32313333	1	1	39
21133112	1	1	99	32332133	1	0	53
21133113	1	0	91	32332223	1	--	70
21133131	1	--	89	32332231	1	0	81
21133211	1	0	91	32332232	1	0	77
21133222	1	1	60	32332322	1	0	72
21133223	1	--	89	32332323	1	0	54
21133232	1	1	81	32332331	1	0	65
21133233	1	0	69	32332332	1	0	62

21133312	1	1	100	32332333	1	1	33
21133313	1	0	71	32333223	1	--	55
21133321	1	--	104	32333232	1	1	63
21133322	1	--	76	32333233	1	--	34
21133331	1	0	85	32333312	1	--	65
21133332	1	0	82	32333323	1	0	39
21211223	1	0	82	32333333	1	--	18
21211321	1	--	87	33113111	1	--	64
21212111	1	0	57	33113211	1	0	84
21212112	1	1	83	33113212	1	--	93
21212122	1	--	59	33113233	1	--	46
21212123	1	0	88	33131333	1	--	25
21212133	1	1	107	33133131	1	--	61
21212212	1	1	63	33133333	1	--	9
21212213	1	0	92	33212213	1	1	77
21212231	1	0	88	33212311	1	--	91
21212321	1	0	83	33212333	1	0	43
21212332	1	1	76	33213211	1	--	93
21212333	1	0	87	33213232	1	--	73
21213121	1	0	83	33213333	1	0	28
21213212	1	1	78	33232233	1	--	38
21213223	1	0	83	33232312	1	--	69
21213231	1	0	97	33232333	1	--	22
21213321	1	0	98	33233131	1	--	59
21213332	1	1	91	33233233	1	--	23
21222112	1	0	61	33233333	1	--	7
21231233	1	0	83	33311231	1	--	85
21232112	1	0	80	33312133	1	0	56
21232122	1	--	56	33312322	1	--	86
21232132	1	0	77	33312333	1	0	36
21232212	1	0	60	33313233	1	0	37
21232222	1	0	36	33313323	1	--	42
21232231	1	0	85	33313333	1	0	21
21232232	1	0	57	33331232	1	--	61
21232233	1	0	82	33331233	1	--	32
21232321	1	0	80	33331333	1	--	16
21232322	1	0	52	33332131	1	--	67
21232323	1	0	81	33332133	1	--	35
21232332	1	0	73	33332232	1	--	60
21233111	1	0	80	33332233	1	--	31
21233132	1	0	92	33332323	1	--	36
21233212	1	1	75	33332333	1	0	15
21233221	1	--	79	33333231	1	0	48
21233233	1	0	67	33333232	1	--	45
21233312	1	--	91	33333233	1	--	16
21233321	1	--	95	33333323	1	--	21
21311122	1	1	80	33333333	1	0	0
21311223	1	0	89	11313223	0*	--	101
21311331	1	0	81	11333323	0*	--	69
21312111	1	0	57	12212223	0*	--	85

21312112	1	1	85	12331221	0*	--	91
21312122	1	1	66	12332123	0*	--	108
21312123	1	0	95	13132333	0*	--	58
21312211	1	0	77	13311311	0*	--	43
21312222	1	1	46	13331123	0*	--	91
21312223	1	0	75	13331333	0*	--	50
21312321	1	0	90	13332332	0*	--	78
21312322	1	1	62	13333123	0*	--	75
21312323	1	0	91	13333223	0*	--	71
21313111	1	0	59	13333233	0*	--	50
21313112	1	1	87	13333323	0*	--	55
21313122	1	1	81	21113222	0*	--	63
21313221	1	0	89	21133123	0*	--	94
21313231	1	1	97	21233322	0*	--	67
21313311	1	0	79	21312121	0*	--	81
21313312	1	1	101	21331112	0*	--	92
21313313	1	0	83	22232213	0*	--	72
21313322	1	1	77	23231121	0*	--	99
21331223	1	0	86	23232221	0*	--	65
21331332	1	0	89	23313322	0*	--	78
21331333	1	0	60	23333122	0*	--	79
21332111	1	0	78	31131323	0*	--	60
21332112	1	0	87	31212132	0*	--	103
21332113	1	0	97	31233111	0*	--	80
21332122	1	0	63	32111211	0*	--	71
21332131	1	0	96	32212122	0*	--	72
21332212	1	0	67	32232222	0*	--	49
21332221	1	0	71	32333322	0*	--	68
21332223	1	0	72	33112112	0*	--	90
21332231	1	0	92	33133323	0*	--	30
21332233	1	0	75	33232322	0*	--	72
21332312	1	0	83	33331223	0*	--	53
21332322	1	0	59	33331321	0*	--	69
21332323	1	--	80	33332221	0*	--	84
21332331	1	0	91	33333321	0*	--	53
21332332	1	0	80	11112123	0	--	70
21332333	1	--	59	11113223	0	--	92
21333111	1	0	80	11132223	0	--	108
21333122	1	0	78	11132232	0	--	100
21333131	1	0	96	11132323	0	--	93
21333132	1	--	93	11231232	0	--	96
21333211	1	0	100	11232323	0	--	91
21333212	1	1	82	11331123	0	--	86
21333221	1	0	86	11332221	0	--	88
21333223	1	0	81	11333122	0	--	98
21333233	1	0	60	12133223	0	--	98
21333312	1	0	91	12211222	0	--	70
21333321	1	0	97	12231231	0	--	85
21333333	1	1	44	12233112	0	--	91
22111223	1	0	74	12233323	0	--	80

22111332	1	1	82	12311333	0	--	89
22112111	1	--	65	12331211	0	--	67
22112131	1	0	83	13132223	0	--	95
22112211	1	0	83	13132322	0	--	96
22112223	1	0	60	13133323	0	--	64
22112321	1	--	75	13212122	0	--	89
22112323	1	0	76	13212321	0	--	81
22112331	1	0	96	13231332	0	--	86
22113111	1	0	67	13232123	0	--	97
22113211	1	--	87	13232311	0	--	78
22113223	1	0	75	13232323	0	--	77
22113232	1	1	67	13233133	0	--	61
22113233	1	0	94	13233233	0	--	57
22113313	1	--	96	13233322	0	--	91
22113323	1	0	91	13332223	0	--	86
22113332	1	1	83	13333222	0	--	93
22131221	1	0	70	21111112	0	--	62
22132112	1	1	72	21111212	0	--	82
22132113	1	0	101	21111312	0	--	82
22132212	1	1	52	21111322	0	--	78
22132231	1	0	77	21131111	0	--	55
22132233	1	0	78	21131212	0	--	83
22132312	1	--	68	21131321	0	--	99
22132323	1	0	73	21132121	0	--	93
22132331	1	0	93	21133212	0	--	84
22133112	1	1	87	21211113	0	--	75
22133211	1	0	95	21211122	0	--	73
22133212	1	1	67	21211211	0	--	63
22133232	1	1	64	21211222	0	--	53
22133312	1	0	83	21233112	0	--	95
22133322	1	1	59	21311111	0	--	43
22133323	1	1	78	21311311	0	--	63
22212111	1	0	74	21311333	0	--	81
22212123	1	0	71	21312212	0	--	70
22212131	1	0	91	21331111	0	--	64
22212212	1	1	46	21331131	0	--	82
22212232	1	1	43	21331312	0	--	97
22212312	1	--	62	21331313	0	--	78
22212321	1	0	66	21333121	0	--	104
22212322	1	1	38	21333133	0	--	64
22213111	1	--	76	21333331	0	--	76
22213112	1	0	81	21333332	0	--	73
22213132	1	1	78	22111231	0	--	89
22213212	1	1	61	22113132	0	--	87
22213222	1	1	37	22121331	0	--	88
22213223	1	0	66	22131111	0	--	72
22213233	1	0	87	22131112	0	--	86
22213312	1	0	77	22131132	0	--	83
22213321	1	--	81	22131133	0	--	93
22222121	1	0	48	22131222	0	--	42

22222222	1	1	0	22131321	0	--	86
22231221	1	0	61	22211132	0	--	77
22231223	1	--	62	22211222	0	--	36
22232112	1	--	63	22211331	0	--	98
22232121	1	0	67	22211332	0	--	73
22232122	1	0	39	22231113	0	--	106
22232123	1	0	68	22231122	0	--	53
22232212	1	1	43	22231211	0	--	85
22232222	1	1	19	22231212	0	--	57
22232223	1	0	48	22231232	0	--	54
22232232	1	1	40	22231311	0	--	101
22232233	1	0	69	22231312	0	--	73
22232311	1	0	87	22231333	0	--	71
22232312	1	0	59	22233331	0	--	87
22232322	1	0	35	22233333	0	--	55
22232323	1	--	64	22311121	0	--	84
22232331	1	0	84	22311212	0	--	67
22232333	1	0	70	22311231	0	--	92
22233112	1	1	78	22311332	0	--	80
22233211	1	0	86	22313333	0	--	69
22233212	1	1	58	22331121	0	--	88
22233221	1	0	62	22331133	0	--	84
22233222	1	--	34	22331211	0	--	92
22233223	1	--	63	22331212	0	--	64
22233312	1	--	74	22331222	0	--	40
22233323	1	0	76	22331321	0	--	84
22233332	1	--	71	22333111	0	--	97
22311123	1	0	92	23111112	0	--	76
22312111	1	0	74	23111212	0	--	87
22312123	1	0	78	23112123	0	--	98
22312132	1	1	70	23112222	0	--	49
22312133	1	0	99	23131332	0	--	84
22312211	1	0	81	23132322	0	--	62
22312221	1	--	57	23133312	0	--	86
22312222	1	1	29	23211121	0	--	81
22312223	1	0	58	23211131	0	--	75
22312231	1	0	78	23211222	0	--	54
22312232	1	1	50	23211311	0	--	77
22312311	1	0	94	23211332	0	--	91
22312312	1	--	69	23212312	0	--	80
22312322	1	1	45	23213231	0	--	104
22312332	1	0	66	23231212	0	--	75
22312333	1	0	84	23231323	0	--	74
22313122	1	1	64	23232223	0	--	66
22313212	1	0	68	23233322	0	--	68
22313221	1	0	72	23311112	0	--	85
22313222	1	--	44	23311221	0	--	89
22313232	1	1	65	23311222	0	--	61
22313233	1	0	85	23311313	0	--	85
22313323	1	1	89	23313121	0	--	97

22313331	1	0	101	23313231	0	--	99
22313332	1	0	81	23331112	0	--	102
22323313	1	0	85	23331212	0	--	82
22331123	1	0	89	23331232	0	--	79
22331221	1	--	68	23332322	0	--	60
22331223	1	0	69	31112121	0	--	72
22331323	1	0	85	31113321	0	--	94
22331332	1	0	77	31131312	0	--	86
22332112	1	1	70	31132221	0	--	103
22332113	1	0	99	31132223	0	--	75
22332121	1	0	74	31132311	0	--	88
22332123	1	0	75	31132312	0	--	85
22332132	1	1	67	31133112	0	--	90
22332211	1	0	78	31133123	0	--	64
22332221	1	0	54	31133212	0	--	86
22332222	1	1	26	31211122	0	--	95
22332223	1	0	55	31211132	0	--	89
22332232	1	--	47	31211211	0	--	63
22332233	1	0	76	31211312	0	--	91
22332312	1	0	66	31212113	0	--	89
22332321	1	--	70	31212211	0	--	77
22332322	1	0	42	31213122	0	--	104
22332332	1	0	63	31213223	0	--	79
22333112	1	0	85	31231211	0	--	84
22333122	1	0	61	31231311	0	--	84
22333131	1	0	100	31231323	0	--	58
22333132	1	0	82	31232322	0	--	82
22333133	1	0	68	31233122	0	--	91
22333211	1	0	93	31233133	0	--	41
22333212	1	1	65	31233222	0	--	81
22333221	1	0	69	31311112	0	--	71
22333222	1	--	41	31311122	0	--	95
22333223	1	0	70	31311212	0	--	91
22333231	1	0	90	31311312	0	--	91
22333311	1	0	98	31312221	0	--	101
22333313	1	--	66	31313123	0	--	76
22333321	1	0	85	31313222	0	--	91
22333323	1	--	69	31313321	0	--	88
22333332	1	0	77	31331221	0	--	99
23112213	1	0	102	31331222	0	--	87
23112221	1	0	77	31331223	0	--	67
23112223	1	0	78	31331332	0	--	59
23112233	1	0	91	31332112	0	--	96
23112323	1	0	94	31332132	0	--	78
23112333	1	0	75	31332221	0	--	98
23113111	1	0	64	31333112	0	--	81
23113112	1	0	92	31333222	0	--	80
23113121	1	0	88	31333223	0	--	51
23113131	1	0	82	31333232	0	--	59
23113212	1	--	88	31333311	0	--	64

23113311	1	0	84	32111112	0	--	79
23113312	1	0	104	32111121	0	--	75
23113323	1	0	81	32111123	0	--	105
23113332	1	0	89	32111311	0	--	71
23122212	1	--	51	32112232	0	--	82
23131323	1	--	76	32112311	0	--	85
23132111	1	0	83	32113112	0	--	95
23132121	1	--	94	32131212	0	--	96
23132212	1	--	70	32131311	0	--	92
23132221	1	0	74	32132211	0	--	106
23132231	1	0	95	32132212	0	--	82
23132232	1	0	67	32132221	0	--	86
23132233	1	0	70	32133113	0	--	65
23132311	1	0	103	32133122	0	--	93
23132323	1	--	75	32133212	0	--	90
23133112	1	--	105	32133223	0	--	64
23133113	1	0	77	32133311	0	--	77
23133121	1	0	109	32133312	0	--	74
23133233	1	0	55	32133321	0	--	80
23133311	1	0	89	32133323	0	--	48
23133321	1	--	92	32211111	0	--	60
23133331	1	0	71	32211112	0	--	88
23133333	1	--	39	32211133	0	--	82
23211132	1	--	95	32211212	0	--	90
23212112	1	--	84	32212111	0	--	74
23212211	1	0	91	32212311	0	--	94
23212212	1	0	64	32213311	0	--	96
23212221	1	0	68	32213312	0	--	93
23212222	1	0	40	32231122	0	--	83
23212231	1	0	89	32231222	0	--	63
23212332	1	--	77	32231332	0	--	70
23212333	1	0	73	32232111	0	--	95
23213112	1	0	99	32232133	0	--	60
23213121	1	0	97	32232212	0	--	73
23213123	1	--	99	32232213	0	--	74
23213211	1	0	93	32232221	0	--	77
23213212	1	1	79	32233112	0	--	92
23213223	1	--	84	32233122	0	--	84
23213232	1	0	76	32233123	0	--	66
23213311	1	0	93	32233232	0	--	70
23213322	1	--	71	32233233	0	--	41
23213333	1	0	58	32311132	0	--	104
23231233	1	0	69	32311212	0	--	97
23232113	1	--	90	32311221	0	--	101
23232131	1	0	104	32311322	0	--	89
23232211	1	0	89	32313122	0	--	94
23232212	1	--	61	32313212	0	--	98
23232233	1	0	68	32313222	0	--	74
23232311	1	--	102	32331111	0	--	81
23232323	1	--	73	32331112	0	--	101

23233212	1	0	76	32331122	0	--	90
23233221	1	--	80	32331132	0	--	83
23233231	1	0	85	32331212	0	--	94
23233232	1	--	73	32331221	0	--	98
23233312	1	--	84	32331222	0	--	70
23233333	1	--	37	32331311	0	--	84
23311233	1	--	83	32331313	0	--	52
23311323	1	0	88	32332111	0	--	95
23312111	1	0	71	32332112	0	--	100
23312112	1	0	91	32332123	0	--	74
23312121	1	0	95	32332211	0	--	99
23312122	1	--	67	32332212	0	--	80
23312123	1	0	96	32332222	0	--	56
23312131	1	0	89	32333122	0	--	88
23312223	1	--	76	32333212	0	--	81
23312311	1	0	91	32333311	0	--	68
23312312	1	0	87	33111122	0	--	100
23312323	1	0	87	33111212	0	--	96
23313111	1	0	73	33111222	0	--	93
23313133	1	--	71	33111312	0	--	93
23313212	1	0	86	33113121	0	--	88
23313222	1	0	62	33113223	0	--	67
23313232	1	0	83	33131122	0	--	95
23313233	1	0	67	33131123	0	--	66
23313323	1	0	72	33131332	0	--	54
23313333	1	0	51	33132222	0	--	76
23331233	1	0	62	33132223	0	--	61
23331323	1	--	67	33132322	0	--	74
23332112	1	0	88	33133121	0	--	82
23332221	1	0	72	33133223	0	--	46
23332222	1	--	44	33133233	0	--	25
23332223	1	0	73	33133321	0	--	62
23332231	1	0	93	33133332	0	--	38
23332311	1	0	95	33211112	0	--	85
23332323	1	0	66	33211211	0	--	77
23332331	1	0	77	33211312	0	--	91
23333111	1	0	94	33211321	0	--	97
23333123	1	--	71	33212222	0	--	70
23333131	1	0	82	33212312	0	--	90
23333211	1	--	96	33212313	0	--	61
23333212	1	0	83	33213112	0	--	95
23333213	1	0	64	33231212	0	--	86
23333222	1	--	59	33231221	0	--	92
23333223	1	0	67	33231312	0	--	70
23333232	1	0	75	33231333	0	--	23
23333233	1	0	46	33232112	0	--	89
23333312	1	0	77	33232122	0	--	87
23333323	1	--	51	33232123	0	--	63
31111233	1	--	76	33232222	0	--	67
31112231	1	0	86	33232223	0	--	59

31112333	1	1	59	33232323	0	--	43
31113131	1	1	68	33233112	0	--	74
31113132	1	1	93	33233221	0	--	76
31113222	1	--	93	33233222	0	--	73
31113323	1	--	65	33233223	0	--	44
31113331	1	1	76	33233323	0	--	28
31113332	1	--	73	33311122	0	--	107
31131233	1	--	55	33311223	0	--	74
31132231	1	0	86	33311311	0	--	77
31132232	1	0	83	33311312	0	--	84
31132333	1	--	38	33311322	0	--	87
31133233	1	--	39	33311332	0	--	66
31133331	1	--	55	33312233	0	--	52
31211131	1	0	61	33312312	0	--	83
31211232	1	0	103	33312323	0	--	57
31212112	1	--	85	33313122	0	--	91
31212212	1	0	93	33313223	0	--	58
31212232	1	0	90	33313311	0	--	71
31212321	1	0	101	33331122	0	--	86
31212323	1	0	78	33331133	0	--	36
31212331	1	1	89	33331311	0	--	66
31212332	1	0	86	33331331	0	--	48
31212333	1	1	57	33332112	0	--	82
31213232	1	--	87	33332121	0	--	88
31213233	1	1	58	33332123	0	--	56
31213323	1	--	63	33332211	0	--	81
31213331	1	1	74	33332223	0	--	52
31213332	1	1	71	33333133	0	--	20
31232231	1	--	84	33333222	0	--	66
31232312	1	--	83	33333223	0	--	37
31232332	1	0	65	33333311	0	--	50
31232333	1	0	36	33333332	0	--	29
31233221	1	--	90				

[1]Sequence listed as parent sequence (A1 = 1, A2 =2, A3 = 3) at each block.

[2]Folding state is 1 for P450s and 0 for no P450; * signifies significant P420 peak.

[3]Chimeras with at least 25% of total product formed by A1 in 30 minutes are active = 1, not active = 0, -- = did not meet minimum P450 concentration requirement of 500 nM.

[4]m is number of sequence changes relative to the closest parent sequence.
