# Supplementary material for: Structure-Guided Recombination Creates an Artificial Family of Cytochromes P450
Source: PLoS Biol. 2006 Apr 11;4(5):e112. doi: 10.1371/journal.pbio.0040112 (PMC1431580; doi:10.1371/journal.pbio.0040112)
Supplement: Supplementary file 4 [file pbio.0040112.st002.pdf]

Table S2. ClustalW amino acid sequence alignment of the heme domain of CYP102A1 (residues 1-463), CYP102A2 and CYP102A3 (residues 1-466). Numbering is according to the CYP102A1 sequence.

```
           1                                                          60
CYP102A1  .TIKEMPQPKTFGELKNLPLLNTDKPVQALMKIADELGEIFKFEAPGRVTRYLSSQRLIKE
CYP102A2  KETSPIPQPKTFGPLGNLPLIDKDKPTLSLIKLAEEQGPIFQIHTPAGTTIVVSGHELVKE
CYP102A3  KQASAIPQPKTYGPLKNLPHLEKEQLSQSLWRIADELGPIFRFDFPGVSSVFVSGHNLVAE

          61                                                         121
CYP102A1  ACDESRFDKNLSQALKFVRDFAGDGLATSWTHEKNWKKAHNILLPSFSQQAMKGYHAMMVD
CYP102A2  VCDEERFDKSIEGALEKVRAFSGDGLATSWTHEPNWRKAHNILMPTFSQRAMKDYHEKMVD
CYP102A3  VCDEKRFDKNLGKGLQKVREFGGDGLATSWTHEPNWQKAHRILLPSFSQKAMKGYHSMMLD

          122                                                        183
CYP102A1  IAVQLVQKWERLNADEHIEVPEDMTRLTLDTIGLCGFNYRFNSFYRDQPHPFITSMVRALD
CYP102A2  IAVQLIQKWARLNPNEAVDVPGDMTRLTLDTIGLCGFNYRFNSYYRETPHPFINSMVRALD
CYP102A3  IATQLIQKWSRLNPNEEIDVADDMTRLTLDTIGLCGFNYRFNSFYRDSQHPFITSMLRALK

          184                                                        245
CYP102A1  EAMNKLQRANPDDPAYDENKRQFQEDIKVMNDLVDKIIADRKASGEQ.SDDLLTHMLNGKD
CYP102A2  EAMHQMQRLDVQDKLMVRTKRQFRYDIQTMFSLVDSIIAERRANGDQDEKDLLARMLNVED
CYP102A3  EAMNQSKRLGLQDKMMVKTKLQFQKDIEVMNSLVDRMIAERKANPDENIKDLLSLMLYAKD

          246                                                        307
CYP102A1  PETGEPLDDENIRYQIITFLIAGHETTSGLLSFALYFLVKNPHVLQKAAEEAARVLVDPVP
CYP102A2  PETGEKLDDENIRFQIITFLIAGHETTSGLLSFATYFLLKHPDKLKKAYEEVDRVLTDAAP
CYP102A3  PVTGETLDDENIRYQIITFLIAGHETTSGLLSFAIYCLLTHPEKLKKAQEEADRVLTDDTP


          308                                                        369
CYP102A1  SYKQVKQLKYVGMVLNEALRLWPTAPAFSLYAKEDTVLGGEYPLEKGDELMVLIPQLHRDK
CYP102A2  TYKQVLELTYIRMILNESLRLWPTAPAFSLYPKEDTVIGGKFPITTNDRISVLIPQLHRDR
CYP102A3  EYKQIQQLKYIRMVLNETLRLYPTAPAFSLYAKEDTVLGGEYPISKGQPVTVLIPKLHRDQ

          370                                                        431
CYP102A1  TIWGDDVEEFRPERFENPSAIPQHAFKPFGNGQRACIGQQFALHEATLVLGMMLKHFDFED
CYP102A2  DAWGKDAEEFRPERFEHQDQVPHHAYKPFGNGQRACIGMQFALHEATLVLGMILKYFTLID
CYP102A3  NAWGPDAEDFRPERFEDPSSIPHHAYKPFGNGQRACIGMQFALQEATMVLGLVLKHFELIN

          432                                  463
CYP102A1  HTNYELDIKETLTLKPEGFVVKAKSKKIPLGGIPSPST.
CYP102A2  HENYELDIKQTLTLKPGDFHISVQSRHQEAIHADVQAAE
CYP102A3  HTGYELKIKEALTIKPDDFKITVKPRKTAAINVQRKEQA
```
