# Supplementary material for: Structure-Guided Recombination Creates an Artificial Family of Cytochromes P450
Source: PLoS Biol. 2006 Apr 11;4(5):e112. doi: 10.1371/journal.pbio.0040112 (PMC1431580; doi:10.1371/journal.pbio.0040112)
Supplement: Supplementary file 5 [file pbio.0040112.st003.pdf]

Table S3. Significance of the top six variables identified by logistic regression analysis of cytochrome P450 chimeras.

Variable	p-value[1]
1	1×10^{-12}
5	3×10^{-8}
7	2×10^{-17}
1-7	3×10^{-12}
1-5	0.01
5-8	0.02

[1]p-values were calculated by removing each variable from the model and applying the likelihood ratio test (see Methods).
