# Supplementary material for: Structure-Guided Recombination Creates an Artificial Family of Cytochromes P450
Source: PLoS Biol. 2006 Apr 11;4(5):e112. doi: 10.1371/journal.pbio.0040112 (PMC1431580; doi:10.1371/journal.pbio.0040112)
Supplement: Supplementary file 6 [file pbio.0040112.st004.pdf]

Table S4. Primers used for site-directed recombination of cytochromes P450 heme domains of A1, A2 and A3.

Tag	Site	Tag Sequence (5' to 3')	Primer (5' to 3')	
N	N-term	-	A123-Nf	GGAGGTACCAGATCT*ATCGATGCTTAGGAGGTCATATG*
Tag 1	64	gaaTGAAGTACT**AC**CTGTC**CTCC**GCTAGCTgaa	A1-64f	GTACTACCTGTCCTCCGCTAGCT*gaaTCACGCTTTGATAAAAACTTAAGT*
			A1-64r	AGCGGAGGACAGGTAGTACTTCA*ttcATCGCATGCTTCTTTAATTAGAC*
			A2-64f	GTACTACCTGTCCTCCGCTAGCT*gaaGAACGGTTTGATAAAAGCATTG*
			A2-64r	AGCGGAGGACAGGTAGTACTTCA*ttcATCACAAACCTCTTTCACCA*
			A3-64f	GTACTACCTGTCCTCCGCTAGCT*gaaAAACGCTTTGACAAGAACCT*
			A3-64r	AGCGGAGGACAGGTAGTACTTCA*ttcATCACACACTTCAGCCAC*
Tag 2	122	atcTAAAGGCCT**AC**ATACT**CTCC**GCTAGCAatc	A1-122f	GGCCTACATACTCTCCGCTAGCA*atcGCCGTGCAGCTTGTTC*
			A1-122r	AGCGGAGAGTATGTAGGCCTTTA*gatATCGACCATCATCGCATGAT*
			A2-122f	GGCCTACATACTCTCCGCTAGCA*atcGCTGTTCAGCTCATTCAAAAATG*
			A2-122r	AGCGGAGAGTATGTAGGCCTTTA*gatATCGACCATTTTCTCATGATAGTC*
			A3-122f	GGCCTACATACTCTCCGCTAGCA*atcGCAACCCAGCTGATTC*
			A3-122r	AGCGGAGAGTATGTAGGCCTTTA*gatATCCAGCATCATAGAATGATAGC*
Tag 3	166	tacTGATCGCGA**AC**AATTA**CTCC**GCTAGCTtac	A1-166f	CGCGAACAATTACTCCGCTAGCT*tacCGAGATCAGCCTCATCC*
			A1-166r	AGCGGAGTAATTGTTCGCGATCA*gtaAAAGCTGTTAAAGCGATAGTTAAA*
			A2-166f	CGCGAACAATTACTCCGCTAGCT*tacAGAGAAACGCCCCACC*
			A2-166r	AGCGGAGTAATTGTTCGCGATCA*gtaGTAACTGTTAAAGCGGTAGTTAAA*
			A3-166f	CGCGAACAATTACTCCGCTAGCT*tacCGTGATTCACAGCATCC*
			A3-166r	AGCGGAGTAATTGTTCGCGATCA*gtaAAAGCTGTTGAATCGATAGTTAAA*
	216/217	-	A1-216f	CGAGGTACCgtcgac*AAAATTATTGCAGATCGCAAAGC*
			A1-216r	CCGCTCGAGgtcgac*TAGGTCGTTCATCACCTTG*
			A2-216f	CGAGGTACCgtcgac*AGCATTATTGCAGAG*
			A2-216r	CCGCTCGAGgtcgac*TAACGAAAACATCGTTTG*
			A3-216f	CGAGGTACCgtcgac*AGAATGATAGCGGAGCGAAAG*
			A3-216r	CCGCTCGAGgtcgac*CAGGGAGTTCATGACTTC*
Tag 4	268	acaTGAACGCGT**AC**TTATT**CTCC**GCTAGCTaca	A1-268f	CGCGTACTTATTCTCCGCTAGCT*acaACAAGTGGTCTTTTATCATTTGC*
			A1-268r	AGCGGAGAATAAGTACGCGTTCA*tgtTTCGTGTCCCGCAATTAAG*
			A2-268f	CGCGTACTTATTCTCCGCTAGCT*acaACGAGCGGCCTGCTTT*
			A2-268r	AGCGGAGAATAAGTACGCGTTCA*tgtTTCATGGCCGGCAATC*

			A3-268f<br>A3-268r	CGCGTACTTATTCTCCGCTAGCT*acaACAAGCGGGTTGCTATC*<br>AGCGGAGAATAAGTACGCGTTCA*tgtCTCATGTCCAGCAATTAAAAATG*
Tag 5	328	gctTAAGTATAC**AC**CATCC**CTCC**GCTAGCAgct	A1-328f<br>A1-328r<br>A2-328f<br>A2-328r<br>A3-328f<br>A3-328r	TATACACCATCCCTCCGCTAGCA*gctCCTGCGTTTTCCCTATATG*<br>AGCGGAGGGATGGTGTATACTTA*agcAGTTGGCCATAAGCGC*<br>TATACACCATCCCTCCGCTAGCA*gctCCGGCTTTCAGCCTTTATC*<br>AGCGGAGGGATGGTGTATACTTA*agcTGTCGGCCATAAGCGC*<br>TATACACCATCCCTCCGCTAGCA*gctCCGGCTTTTTCTCTATATGC*<br>AGCGGAGGGATGGTGTATACTTA*agcTGTTGGATACAGTCTGAGG*
Tag 6	404	cagTGATTCGAA**AC**CATTA**CTCC**GCTAGCTcag	A1-404f<br>A1-404r<br>A2-404f<br>A2-404r<br>A3-404f<br>A3-404r	TCGAAACCATTACTCCGCTAGCT*cagTTCGCTCTTCATGAAGC*<br>AGCGGAGTAATGGTTTCGAATCA*ctgCTGACCGATACACGCAC*<br>TCGAAACCATTACTCCGCTAGCT*cagTTTGCCCTTCATGAAGC*<br>AGCGGAGTAATGGTTTCGAATCA*ctgCATGCCGATACAGGCC*<br>TCGAAACCATTACTCCGCTAGCT*cagTTTGCTCTTCAAGAAGCG*<br>AGCGGAGTAATGGTTTCGAATCA*ctgCATGCCAATACAAGCGC*
C	C-term	-	A1-Cr<br>A2-Cr<br>A3-Cr	CAACAATTG*TTAAGTGCTAGGTGAAGGAATACC*<br>CAACAATTG*TTATTCAGCTGCCTGGACGTC*<br>CAACAATTG*TTATGCCTGTTCTTTTCTCTGTAC*

Bold letters: *BsaX*I recognition site; underline: *Nhe*I recognition site; Lower-case letters: consensus sequence (sticky ends by *BsaX*I digestion); Italic letters: complementary to parental gene sequence; Shadowed letters: *Bgl*II or *Mfe*I recognition site of N-terminal or C-terminal primer, respectively.
