# Supplementary material for: Structure-Guided Recombination Creates an Artificial Family of Cytochromes P450
Source: PLoS Biol. 2006 Apr 11;4(5):e112. doi: 10.1371/journal.pbio.0040112 (PMC1431580; doi:10.1371/journal.pbio.0040112)
Supplement: Supplementary file 7 [file pbio.0040112.st005.pdf]

Table S5. Sequences of probes for hybridization analysis.

Block	Parent	Sequence (5' to 3')
1	A1	GAC AAT TAA AGA AAT GCC TCA GCC AAA AAC GTT TGG
	A2	GAA GGA AAC AAG CCC GAT TCC TCA GCC
	A3	GAA ACA GGC AAG CGC AAT ACC TCA GCC
2	A1	GCG CAT AAT ATC TTA CTT CCA AGC TTC AGT CAG C
	A2	GCG CAC AAC ATT CTG ATG CCG ACG TTC
	A3	CC CAC CGC ATT TTG CTG CCG AGT TTT AG
3	A1	GAG CGT CTA AAT GCA GAT GAG CAT ATT GAA GTA CCG
	A2	GCA AGG CTC AAC CCG AAT GAA GCA GTC
	A3	G AGC CGG TTA AAC CCC AAT GAA GAA ATT GAT GTA G
4	A1	CCA GAC GAC CCA GCT TAT GAT GAA AAC AAG C
	A2	GTT CAA GAT AAG CTT ATG GTC AGA ACA AAG CGG C
	A3	CTG CAA GAT AAA ATG ATG GTG AAA ACG AAG CTG CAG
5	A1	GCA AGC GGT GAA CAA AGC GAT GAT TTA TTA ACG C
	A2	GCG AAT GGA GAC CAG GAT GAA AAA GAT TTG CTC
	A3	GCG AAT CCG GAT GAA AAC ATT AAG GAT CTC TTG TC
6	A1	GCA GCA CGA GTT CTA GTA GAT CCT GTT CC
	A2	GTC GAT CGG GTG CTG ACG GAT GCA G
	A3	GCG GAT CGC GTG TTA ACG GAT GAC AC
7	A1	CCA AGT GCG ATT CCG CAG CAT GCG
	A2	CAG GAC CAA GTG CCT CAT CAT GCG TAC
	A3	CCT TCA AGT ATC CCT CAC CAT GCG TAT AAG C
8	A1	ATC GAA AAA AAT TCC GCT TGG CGG TAT TCC TTC AC
	A2	GCC GTC ATC AGG AAG CCA TTC ATG CAG A
	A3	CGC GAA AAA CAG CGG CAA TCA ATG TAC AGA G
